# COVID-19 During Development: A Matter of Concern

**DOI:** 10.3389/fcell.2021.659032

**Published:** 2021-04-07

**Authors:** Lucas Paulo Jacinto Saavedra, Kelly Valério Prates, Gessica Dutra Gonçalves, Silvano Piovan, Paulo Matafome, Paulo Cezar de Freitas Mathias

**Affiliations:** ^1^Laboratory of Secretion Cell Biology, Department of Biotechnology, Genetics and Cell Biology, State University of Maringa, Maringa, Brazil; ^2^Institute of Physiology and Institute of Clinical and Biomedical Research, Faculty of Medicine and Center for Innovative Biomedicine and Biotechnology, University of Coimbra, Coimbra, Portugal; ^3^Coimbra Health School, ESTeSC, Instituto Politécnico de Coimbra, Coimbra, Portugal; ^4^Clinical Academic Center of Coimbra, Coimbra, Portugal

**Keywords:** COVID-19, cardiometabolic disease, SARS-CoV-2, development, metabolic programming, public health

## Abstract

A new infectious disease, COVID-19, has spread around the world. The most common symptoms of severe acute respiratory syndrome coronavirus 2 (SARS-CoV-2) infection are cough and fever, but severe cases can develop acute respiratory distress syndrome. The main receptor for SARS-CoV-2 in human tissue is angiotensin-converting enzyme 2, and the lungs, heart, and kidneys are the most affected organs. Besides the inflammatory process and tissue damage, the presence of a cytokine “storm” has been related to a higher mortality rate. Other infectious viral diseases, such as Zika, chikungunya, and influenza, were associated with complications in pregnant women, such as growth restriction, malformation, preterm birth, low birth weight, miscarriage, and death, although they can also cause developmental disorders in infants and adolescents. Evidence points out that stressors during pregnancy and infancy may lead to the development of obesity, diabetes, and cardiovascular disease. Therefore, we hypothesize that COVID-19 infection during the critical phases of development can program the individual to chronic diseases in adulthood. It is important that COVID-19 patients receive proper monitoring as a way to avoid expensive costs to public health in the future.

## Introduction

Severe acute respiratory syndrome coronavirus 2 (SARS-CoV-2) is the strain of the newly discovered infectious disease COVID-19. Its high level of contagion led to a global outbreak at the start of 2020, and it has to date reached 219 countries or territories (WHO, 2021). The early symptoms are mild, usually starting from day 7 of infection, and they can quickly evolve to acute respiratory distress syndrome (ARDS; Chavez et al., 2020). Respiratory system damage is the main factor leading to lethality. However, the infection can affect other organs, such as the heart, kidneys, and liver, in addition to the immune and circulatory cells (Wang et al., 2020). Without a vaccine or drug with proven effectiveness, SARS-CoV-2 still can infect millions of people, extending restrictions measures indefinitely (Kissler et al., 2020).

The current pandemic will change the way that new generations will live, both in the socioeconomic and health fields. Despite the immediate effects of COVID-19, studies have shown physiologic changes during pregnancy that will affect future generations, including the increase in fatality rate, decreased tolerance to hypoxia during pregnancy, and fetal distress (Assiri et al., 2016; Liu et al., 2020; Mullins et al., 2020). It is thus necessary to investigate the impacts of the changes caused by COVID-19 during stages of development, such as pregnancy, lactation, and adolescence, toward preventing their consequences in adult life. People who have recovered from infectious diseases can have permanent damages; for example, pneumonia can result in permanent damage to lung tissue (Sun et al., 2020). The monitoring of patients who have recovered from the infection will allow the formulation of plans that aim to reduce the expenses with treatments, avoiding a collapse of the health system. All in all, all lives will be affected by this virus. This article proposes a hypothesis where COVID-19 infection during the critical phases of development, such as in infancy and adolescence, may increase the risk of cardiometabolic disease in the next generations, further increasing the public health expenses in the future.

## Outbreak of Coronavirus and Emerging Infectious Diseases

The novel SARS-CoV-2 virus has disseminated all over the world and has caused a broad spectrum of manifestations, ranging from asymptomatic cases to pneumonia and even death. These unprecedented times originated in late 2019 in Wuhan, China, and ongoing scientific research is trying to find out how the coronavirus has passed on to humans (Shereen et al., 2020). A few routes are possible, such as through blood, urine, and, more likely, a possible direct contact between the original wildlife animal and humans. However, none of these have yet been proven. Analysis of the receptor-binding domain, which is an essential part of coronavirus that allows it to hold on to and invade a cell, indicates that SARS-CoV from bats and SARS-CoV-2 have the most genetic similarity (96%; Andersen et al., 2020; Li X. et al., 2020). On the other hand, a direct transmission from bats to humans seems unlikely because of the absence of contact. Therefore, it has been suggested that a pangolin might be a possible intermediate host of the virus to humans (Lam et al., 2020) Figure 1.

**FIGURE 1:**
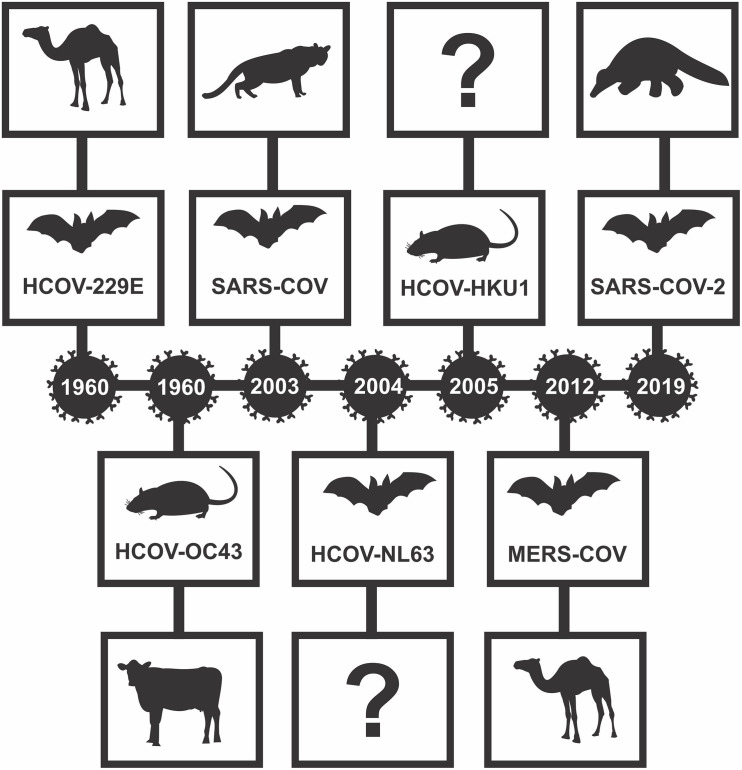
Human Important Coronaviruses Timeline. 1960 – HCOV-229E emerged a progenitor virus found in bat and intermediary host camelids; HCOV-OC43 originated in rats and has as intermediary host cows. 2003 – SARS-CoV emerged from bat viruses with civets as intermediary hosts. 2004 – HCOV-NL63 emerged from bats with an unknown intermediary host. 2005 – HCOV-HKU1 emerged from rats with an unknown intermediary host. 2012 – MERS-COV emerged from bats, with camels as intermediary host. 2019 – SARS-CoV-2 emerged from bats with pangolin as the intermediary host. Adapted from Cui et al. (2019) and Mittal et al. (2020).

The first version of severe acute respiratory syndrome (SARS) appeared in 2003, also in China, and it caused hundreds of deaths (Zhong et al., 2003), as seen in Figure 1. Another example of a previous virus outbreak is the Hendra virus, starting in 2016 in Australia, and it caused both human and horse deaths (Wang and Anderson, 2019). Ebola is also an infectious disease that is likely to have bats as natural reservoir hosts. It was first reported in 1976; however, between 2013 and 2015 it spread out to several West African countries, and it is probable that its transmission occurs through blood-to-blood and secretions contact (Hranac et al., 2019). Scientific data shows so far that there are many challenges ahead, especially those involving emerging infectious diseases. SARS-CoV-2 is highly contagious and has a rapid spread and persistent infection, which may lead to serious complications in several people. As other viruses, it is more contagious in symptomatic individuals (Li X. et al., 2020). It is believed that this spread is similar to that of other coronaviruses, i.e., by close human-to-human contact and respiratory droplets but also through surfaces and fomites containing the virus and contact with mucosa (Andersen et al., 2020).

## SARS-CoV-2 Infection and Disease

**FIGURE 2:**
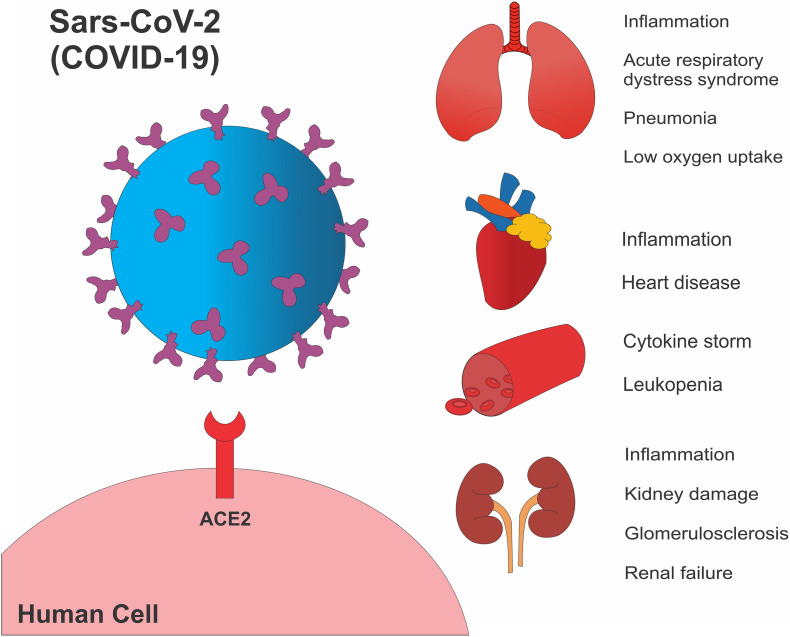
SARS-CoV-2 cell infection and organ damage. The viral Spike Glycoprotein is able to infect human cells by binding to the angiotensin-converting enzyme 2 receptor, thereby diffusing through the cell membrane and infecting the cell. It is also demonstrated pathological findings and complications of different organs affected by the disease.

In most COVID-19 cases, elderly and individuals with pre-existing comorbidities showed an increased susceptibility to developing complications of COVID-19 disease; there was a higher case-fatality rate of 14,8% for individuals aged 80 years and older, 10,5% for cardiovascular disease, 7,3% for chronic respiratory disease, 7,30% for diabetes, and 6,0% for hypertension (Wu and McGoogan, 2020). Moreover, emerging data has been pointing to the potential risk for obese individuals, which may have seen 2.42-fold higher odds of developing severe pneumonia by SARS-CoV-2 (Qingxian et al., 2020). The average incubation period for the SARS-CoV-2 virus is 5.2 days, varying from 2 to 14 days, and the most common symptoms by SARS-CoV-2, after the incubation period, are cough and fever (Sohrabi et al., 2020). However, there are several individuals presenting muscle soreness, fatigue, and ARDS, with the latter characterizing the severe form of the disease (Chavez et al., 2020). SARS-CoV-2 infection occurs through the binding of the Spike Glycoprotein (S) of the viral surface with angiotensin-converting enzyme 2 (ACE2), highly expressed in pneumocytes (Walls et al., 2020; Figure 2). Next, the S1 domain of the S protein is responsible for attaching the virus into the ACE2 and the cellular serine protease TMRPSS2cleaves S1/S2 site, allowing for viral fusion into the cell membrane to its entrance into the lung cells (Hoffmann et al., 2020; South et al., 2020; Walls et al., 2020). Curiously, besides old age and the presence of metabolic disease, the presence of higher blood cytokine levels was also related to the severity of COVID-19. After the virus enters the host cell through endocytosis, it starts to replicate, leading to events such as a higher protein synthesis stress, viral RNA recognition, and apoptosis, which in turn promotes a chemokine surge (Kakodkar et al., 2020). Regarding the severe form of the disease, it is believed that an aggressive increase in pro-inflammatory cytokines, which have been called a “cytokine storm,” is strongly related to lung injury, ARDS, multi-organ failure, and death (Prompetchara et al., 2020; Figure 2). Recently, animal studies have been pointing to a direct role in cytokine storm and multi-organ injury. It was shown that TNFα and IFNγ play a critical role in inducing PANoptosis—an inflammatory programmed cell death, ultimately leading to organ injury (Karki et al., 2021).

Organs that were shown to be sensitive to SARS-CoV-2 infection also commonly have high levels of ACE2 in the cell membrane (Clerkin et al., 2020; Diao et al., 2020; Larsen et al., 2020; Zhang et al., 2020). Impairment of cardiac function was associated with mononuclear inflammatory infiltration in individuals who died due to COVID-19 (Xu et al., 2020). Also, increased blood levels of troponin were related to an increase of IL-6, ferritin, and lactate dehydrogenase, typifying a secondary hemophagocytic lymph histiocytosis in those individuals with COVID-19 (Clerkin et al., 2020). In the kidney, the presence of acute kidney injury occurred in 5.1% of the 701 hospitalized people by COVID-19 in Wuhan, China, and it was related to the increased baseline serum creatinine and blood urea nitrogen in addition to a high association between the presence of kidney injury and death (Cheng et al., 2020). In the liver, the presence of liver injury marks, such as alanine aminotransferase (ALT) and aspartate aminotransferase (AST), together with reduced platelet counts and albumin, was related to higher mortality among COVID-19 patients (Boettler et al., 2020; Guan et al., 2020; Zhang et al., 2020). Thus, understanding how the SARS-CoV-2 cause damages in the organism, and the early detection of clinical features such as elevated cytokines, abnormal kidney and liver function, anemia, and lymphopenia, can lead to correct treatment of the patient, which also can prevent the development of complications by SARS-CoV-2 and the irreversible damages in other organs and systems.

## Infectious Diseases During Critical Phases of Development and Next-Generation Outcomes

Physiological changes occurring during pregnancy are adaptations for optimal fetal development. Together with infancy and adolescence, pregnancy is the life phase more studied as a critical phase of development, where the individual may be shaped by a wide variety of intrinsic or extrinsic (environmental) factors (Burggren and Mueller, 2015). According to Barker, the physiological dysfunctions caused by insults can go throughout life phases and be responsible for the development of health or disease in adulthood (Barker and Osmond, 1986; Barker et al., 2002; Bateson et al., 2004).

### Pregnancy

Human and experimental data have pointed out that, not only pregnancy increases the risk for a variety of infectious diseases, such as pneumonia, but also viral infections are associated with poorer outcomes of offspring (Visscher and Visscher, 1971). During pregnancy, an altered immune function is observed, such as an increased generalized inflammatory response from inmate immunity and a down-regulation of T-cells function, representing a decreased adaptative immunity (Luppi, 2003). It is believed that, at least in part, the effects observed on adaptative immunity could be modulated by progesterone, which is physiologically increased during this phase (Shah et al., 2018). Collectively, these adaptations from the immune system are essential for the maintenance of pregnancy. In the realm of viral infections during pregnancy, there is plenty of evidence pointing out the poor outcomes for child development. Chikungunya viral infection during pregnancy may be transmitted to the fetus, causing severe fetal infection and encephalitis (Charlier et al., 2017). Dengue, a viral disease common in South America, during pregnancy is associated with preterm birth and low birth weight (Paixao et al., 2016). Zika virus infection during pregnancy is associated with intrauterine growth restriction and the development of microcephaly in the offspring (Hajra et al., 2017).

Given the ongoing pandemic of COVID-19, pneumonia is one of the respiratory diseases affecting people positively tested for this disease. Previous coronaviruses infections, such as SARS and MERS, have been associated with negative outcomes for pregnant women and their babies. Data from the World Health Organization (WHO) estimates that at least 100 pregnant women were infected with SARS during 2002 and 2003 (World Health Organization, 2003). So far, no detection of vertical transmissions has been detected to SARS, MERS, or COVID-19 in pregnant women with or without pulmonary manifestations (Schatz et al., 1990; Robertson et al., 2004; Assiri et al., 2016; Li Y. et al., 2020). Interestingly, antibodies to SARS have been found in cord blood and breast milk (Robertson et al., 2004). A clinical study conducted in Hong Kong, one of the regions that most spread SARS, has shown that 80% of the pregnant SARS patients experienced preterm delivery, complicated pregnancies, and insufficient growth of the fetus (Wong et al., 2004). Data obtained by the review of epidemiological and clinical features of COVID-19 infection during pregnancy found that 37% of women presented preterm delivery and 30% had fetal distress (Zimmermann and Curtis, 2020). A report based on 116 cases observed no association between COVID-19 infection during pregnancy and an increased risk for preterm birth (Yan et al., 2020).

Preterm birth has been associated with an increased risk of developing cardiovascular disease and metabolic syndrome later in life (Liao et al., 2020). In fact, a meta-analysis of the literature has shown that preterm birth was significantly associated with increased fat mass, blood pressure, fasting glucose, and insulinemia, indicating that these individuals are at greater risk of developing cardiometabolic diseases during adulthood (Markopoulou et al., 2019). Lam et al. (2020), have studied 10 pregnant women infected with SARS, also finding an increase in miscarriage, renal failure, and death (Lam et al., 2020).

During pregnancy, there is an increase in oxygen demand (Alaily and Carr, 1978), and hypoxia is a common condition among mothers infected with coronavirus diseases (Assiri et al., 2016), aggravating pregnant prognosis. Regarding pregnant MERS patients, a case report study has shown that mothers either had miscarriages or lost their baby a few hours after delivery (Assiri et al., 2016). Similarly, newborns from mothers positively tested to COVID-19 have shown complicated outcomes, such as edema and the need for oxygen therapy (Chen et al., 2020). During pregnancy, hypoxia is a complication commonly associated with decreased body weight. Indeed, in the United States, low birth weight and premature complications are responsible for approximately 75% of perinatal deaths (Hutter and Jaeggi, 2010; Giussani, 2016). Experimentally, Cuffe et al. (2014) related that the hypoxia in the last part of pregnancy caused morphological changes in mice placenta. Data published by Walton (2017, 2018) showed that maternal hypoxia in the last trimester of gestation in mice induced a decrease in the offspring body weight, as well as alterations in the kidney and blood pressure. Several studies have demonstrated that low birth weight is associated with cardiovascular diseases in adulthood (Barker, 1999), but also type II diabetes and obesity (Barker and Osmond, 1986; Barker, 2005; McDonald et al., 2010; Calkins and Devaskar, 2011). Women who were born with low-birth-weight presented significantly higher glycemia, insulinemia, and prevalence of diabetes and metabolic syndrome (Jornayvaz et al., 2016). A meta-analysis of 135 studies has shown that birth weight is correlated with the risk of developing type II diabetes and cardiovascular disease in a J-shaped manner, with people with low birth weight being at greater risk of development of those diseases (Knop et al., 2018). Thus, the mothers infected by SARS-CoV-2, who presented symptoms such as hypoxia, may have an impairment of fetal development, which may ultimately lead to poor outcomes in the offspring’s metabolic and cardiovascular health.

### Infancy and Adolescence

Epidemiological data points out that children infected with COVID-19 present mild clinical symptoms during the acute infection and are less likely to develop a serious form of the disease when compared to adults and mainly elderly people (Zimmermann and Curtis, 2020). Despite that, recent evidence has shown the development of a late inflammatory response in children infected by COVID-19, named Multi system Inflammatory Syndrome in children (MIS-C; Jiang et al., 2020). It is believed that MIS-C is not directly caused by a viral infection but by the adaptative immunity response triggered, which may lead to increased inflammatory markers, shock, and multiorgan impairment, with possible long-term consequences for cardiovascular morbidity in children with MIS-C (Jiang et al., 2020).

In addition to pregnant women and infants, adolescents can also be affected by viral infections. Although most people who have died from COVID-19 are elderly, the infection can cause sequelae that decrease the quality of life. A study with adolescents infected by SARS demonstrated pulmonary sequels with abnormal lung function and a decrease in exercise performance responses with a persistence of sequelae after 6 months of infection (Li et al., 2004). According to the Center for Diseases Control and Prevention only 0.3 and 0.1% of the hospitalization by COVID-19 are between 0–4 years old and 5–17 year old, respectively; however, it is important to be concerned with the results of these infected children who needed hospitalization (Garg, 2020). Little or nothing is known about the effect of COVID-19 in children and adolescents; however, some studies have been concerned with the mental health of this group since most mental disorders start in childhood (Golberstein et al., 2020).

**FIGURE 3:**
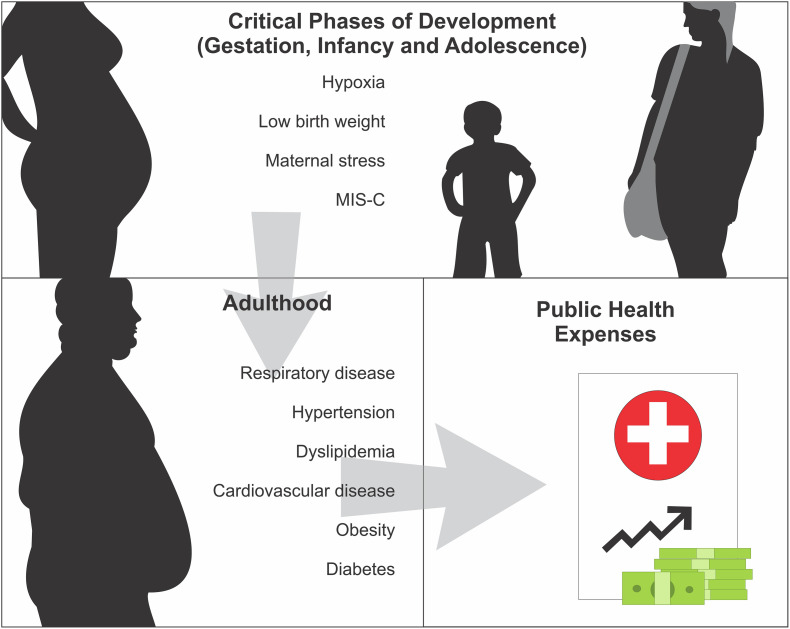
COVID-19 during critical phases of development and future outcomes. Viral infections, including that one caused by COVID-19 during the critical phases of development, may lead to negative outcomes in pregnancy, but also infancy and adolescence, which in turn we hypothesize that can increase the risk to the development of NCD’s during adult life, leading to further public health expenses in the future.

Therefore, viral infections during pregnancy and other programming windows can be an important risk factor for the development of changes in the offspring that may increase the risk for diabetes, obesity, and cardiovascular disease during adult life (Figure 3). It is crucial to understand the pathogenesis of coronaviruses in childbirth and the long-term effect it can have on infant health. Thus, the correct care of these individuals, infected by COVID-19 and with some sequelae, may reduce the public spending on health in the future.

## COVID-19: A Problem of Public Health to the Future

Throughout human history, some viral diseases have caused thousands of deaths, leading to the collapse of the public health system in several countries. Appropriate actions during viral infection may prevent it from spreading and, as result, avoid the high cost to public health. Diseases such as SARS and MERS are coronaviruses and have caused 774 (Chan-Yeung and Xu, 2003) and 586 (Kim et al., 2017) deaths, respectively. During the SARS epidemic, it has been estimated that we have so far seen a loss of $80 billion in the economy due to the breakdown in the economic activities (Lee and McKibbin, 2004). During the SARS outbreak, there was a global response in which organizations created methodologies along with Chinese governance, thereby avoiding the spread of SARS (Fidler, 2004; Lee and McKibbin, 2004). In contrast, during the MERS and Ebola epidemics, the poor strategies associated with a failed hospital infrastructure and no support for isolation during the breakout caused increased mortality with financial loss (Al Shehri, 2015; Rajakaruna et al., 2017). Therefore, the proper approach during the peak of infection may prevent the collapse of public health and decrease hospitalization in cases of countries with poor hospital infrastructure (Vessey and Betz, 2020).

At the moment, due to the increasing transmission rate of SARS-CoV-2, the vulnerability of health systems when faced with the pandemic has become evident; the control of the virus spread is still based on population testing, social distancing, use of masks, and hygiene protocols (Salathe et al., 2020). On the other hand, the population immunization by vaccine injection in several countries is already happening, and vaccines against SARS-CoV-2 currently in use involve inactivated subunits of the virus based on non-replicable adenoviral vectors and mRNA that induces human cells to produce viral proteins (Vora et al., 2020).

In addition to the vulnerability to virus infection, the population that remains without access to the vaccine still faces the economic and social challenges imposed by the restrictions and afflictions of the pandemic. Several studies show the impact on the most vulnerable social classes, and these range from increased unemployment to hunger to compromised mental health (Huang and Zhao, 2020; Werneck et al., 2020). Thus, vaccination proves to be a strategy to contain not only the pandemic but also its socioeconomic effects. Other cases of viral pandemics that have impacted the world socioeconomically and are now controlled by vaccination demonstrate the importance of mass immunization in the population (Sander et al., 2010). Vaccines against diseases such as poliomyelitis, yellow fever, and smallpox have caused a huge cost to the countries of North and South America, e.g., approximately US$ 240 million. However, there was a great reduction of infection cases with lead to a reduction in health public spending estimated at more than US$ 430 million in treatment costs (de Quadros, 2002). Also, children from 2 to 5 years old who are vaccinated against Influenza induced a reduction in public and private spending on treating this and other respiratory infections (White et al., 1999; Cohen and Nettleman, 2000; Esposito et al., 2006).

It is known that women are at greater risk of contracting SARS-CoV-2 infection (Vora et al., 2020). However, studies analyzing the vaccine effects are still under evaluation, and those that have already been completed and approved do not evaluate the consequence according to gender, neither the safety for pregnant nor lactating women, since pregnancy was an exclusion criterion (Vora et al., 2020). Such a fact may expose those born during the pandemic to a greater risk of being affected by the infection since the vaccination is not considered safe for pregnant women. Furthermore, it is already known that ACE2 and TMPRSS2 are expressed in the human placenta and, although only two cases of vertical transmission have been reported, a placental transmission and fetus infection, is possible to occur (Hecht et al., 2020).

Together, correct healthcare of the people who have been infected can prevent expensive spending in the future. Several studies have shown that the right approach in various diseases, not only in infectious diseases but also in non-communicable diseases (NCD), can prevent future expenses for the government. Evidence has shown that, after famines, such as the Dutch famine (Roseboom et al., 2011), Bangladesh famine (Finer et al., 2016), Chinese famine (Zimmet et al., 2018), and World War II (Mink et al., 2020), there was a correlation between the increase in NCD in adults and elderly people who experienced these events during early life, leading to public health expenditures on treatment for these individuals. In this sense, an action plan was developed to decrease NCD, which includes monitoring individuals who were born with or develop alterations during critical periods of life as a means of prevention (Uauy et al., 2011). Respiratory infections were associated with chronic obstructive pulmonary disease, asthma, and cystic fibrosis permanently (Hewitt et al., 2016; Britto et al., 2017). Nevertheless, after 12 years, 25 patients who recovered from SARS still presented with metabolic alterations, such as hyperlipidemia and glucose metabolism disorders (Wu et al., 2017). Regarding this, COVID-19 disease has been related with possibly irreversible pathological findings, causing modifications in organ structures during developmental phases, such as gestation, infancy, and adolescence, which may cause cardiometabolic diseases in adulthood. In order to avoid more expensive costs caused by SARS-CoV-2, as therapeutic interventions, the annual monitoring of adult patients and babies from mothers diagnosed with COVID-19 could reduce the risk to the development of NCD in the future.

## Future Directions

Since late 2019, we have been experiencing a global health crisis. The WHO data have shown that by the beginning of March 2021, more than 113 million people were confirmed to COVID-19 (WHO, 2021). In this matter, the research community has been trying to answer questions, such as: Can discovering how these viruses have passed on to humans prevent future transmissions? Could it lead to irreversible damages to the population? Will the socioeconomic status be able to recover in all regions of the world? In addition, understanding the long-term effects of COVID-19 on human health is crucial to mitigating the impacts on the next generation. There are several case reports studying the impacts of COVID-19 on pregnant women and the damage to organs in people at several stages of life. However, most of those studies have been carried out in China (i.e., little variety) and have limitations, such as the number of subjects and the lack of data showing the health effects of children born to mothers exposed to COVID-19. Regarding the consequences of COVID-19 on human health, data have shown that the main organs affected by SARS-CoV-2 are the lungs, heart, and kidneys. So far, we do know whether people with COVID-19 are more likely to have abnormal immune system responses to several diseases. Despite this, in these unprecedented times, it is impossible to answer how and when the history of COVID-19 will end, further studies are needed to better understand the long-term effects of COVID-19 infection during the critical phases of development in the risk to the development of cardiometabolic diseases, as well as to plan and decrease the cost of treatments for COVID-19 consequences. Despite the vaccination program that is occurring worldwide, that this disease still affects populations and causes outbreaks in all countries together with the fact that new variants of SARS-CoV-2 are emerging has brought about new doubts about future consequences (Fontanet et al., 2021). Taken together, the hypothesis approached in the current study has high relevance and can help us to understand the importance to create preventive methodologies to avoid the possible increase of NCD and health public spending due to future consequences of COVID-19.

## Author Contributions

LS, KP, GG, and SP were responsible for searching, reviewing, andwriting. LS, KP, GG, SP, PMa, and PMh were responsible for preparing the final manuscript and all necessary corrections of this manuscript. All authors contributed to the article and approved the submitted version.

## Conflict of Interest

The authors declare that the research was conducted in the absence of any commercial or financial relationships that could be construed as a potential conflict of interest.
